# Design of a series visco-elastic actuator for multi-purpose rehabilitation haptic device

**DOI:** 10.1186/1743-0003-8-3

**Published:** 2011-01-20

**Authors:** Jakob Oblak, Zlatko Matjačić

**Affiliations:** 1 University Rehabilitation Institute, Republic of Slovenia, Linhartova 51, 1000 Ljubljana, Slovenia

## Abstract

**Background:**

Variable structure parallel mechanisms, actuated with low-cost motors with serially added elasticity (series elastic actuator - SEA), has considerable potential in rehabilitation robotics. However, reflected masses of a SEA and variable structure parallel mechanism linked with a compliant actuator result in a potentially unstable coupled mechanical oscillator, which has not been addressed in previous studies.

**Methods:**

The aim of this paper was to investigate through simulation, experimentation and theoretical analysis the necessary conditions that guarantee stability and passivity of a haptic device (based on a variable structure parallel mechanism driven by SEA actuators) when in contact with a human. We have analyzed an equivalent mechanical system where a dissipative element, a mechanical damper was placed in parallel to a spring in SEA.

**Results:**

The theoretical analysis yielded necessary conditions relating the damping coefficient, spring stiffness, both reflected masses, controller's gain and desired virtual impedance that needs to be fulfilled in order to obtain stable and passive behavior of the device when in contact with a human. The validity of the derived passivity conditions were confirmed in simulations and experimentally.

**Conclusions:**

These results show that by properly designing variable structure parallel mechanisms actuated with SEA, versatile and affordable rehabilitation robotic devices can be conceived, which may facilitate their wide spread use in clinical and home environments.

## Background

Rehabilitation robotics is a rapidly evolving field [1-4]. Numerous haptic robots were developed for movement training of upper extremities following neurological disorder. According to works published to date, robots for upper extremity motor rehabilitation are usually serial linkage mechanisms that can be in general divided in two groups. The first consists of serial linkage mechanisms with only 1 to 3 degrees of freedom (DOF), where the end-effector of the robot is in contact with the user's hand, making it suitable for only one activity of upper extremity movement (either arm reaching movement or wrist movements). Clinical use of such low-DOF serial mechanisms [5-10] necessitates the use of two or more devices in order to provide comprehensive upper extremity movement training. This is neither convenient from a practical nor cost-effective point of view. On the other hand, exoskeleton mechanisms may have up to 7 DOF [11-13] and can provide comprehensive upper extremity movement rehabilitation. However, such mechanisms require high quality back drivable actuators for each DOF, which necessitates complex and thus expensive design.

Few rehabilitation robots have implemented a parallel kinematic structure. Parallel mechanisms usually have mechanical linkages with many DOF that greatly exceed the resulting DOF of the whole mechanism. This property allows for a design where some of the joints may be easily locked or unlocked, thus resulting in different workspace configurations suitable for different aspects of arm or wrist movements training. Another characteristic of parallel robots is that the actuators are located at the robot's base. This feature allows the implementation of series elastic actuators (SEA) [14-16] that utilize standard off-the-shelf DC motors with suitable planetary gearheads and suitable springs, providing similar overall haptic performance as their high quality back-drivable counterparts. Universal Haptic Drive (UHD) [17] and Variable Structure Pantograph (VSP) [18] are the two devices in which mechanisms with lockable joints and SEA actuation were successfully implemented and tested in clinical practice.

However, from a control point of view, both beneficial aspects; parallel kinematic structure (such as VSP) and SEA based drive, result in a mechanical system where the reflected masses of the SEA and the parallel kinematic structure (serially connected with a spring) become comparable, resulting in a coupled mechanical oscillator. Suitable control of such a rehabilitation robot, which should provide stable haptic interaction when in contact with a human, may present a considerable challenge, not addressed in previous studies.

The aim of this paper was to investigate theoretically, through simulations and experimentally the necessary conditions that guarantee stability and passivity of a haptic device, based on a variable structure parallel mechanism driven by SEA actuators, when in contact with a human. We have analyzed an equivalent mechanical system where a dissipative element, a mechanical damper, was placed in parallel with a spring in SEA. The goal was to derive conditions that must be met in order to allow use of a SEA driven variable structure parallel mechanism as a stable haptic interface in upper extremity rehabilitation.

## Methods

### Variable structure pantograph: mechanical linkage

**Figure 1:**
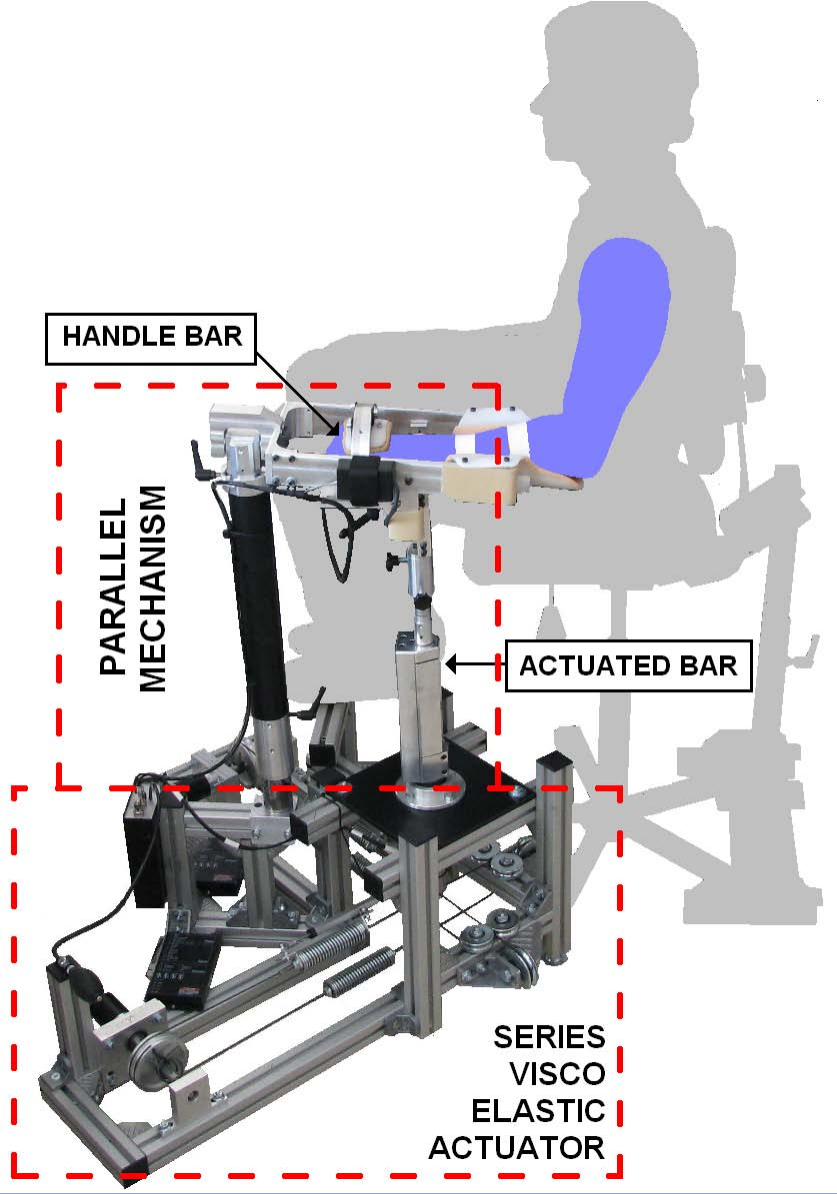
**The photograph of variable structure pantograph (VSP)**. The essence of the VSP is variable structure parallel mechanism, which is driven by a visco-elastic actuator. The VSP promises high suitability for training of upper extremity tasks involving hand positioning and orientation.

Figure 1 shows a VSP haptic device that is composed of a variable structure parallel linkage and two SEA actuators. A brief description on the device is provided here, while more detailed information can be found in [17,18].

**Figure 2:**
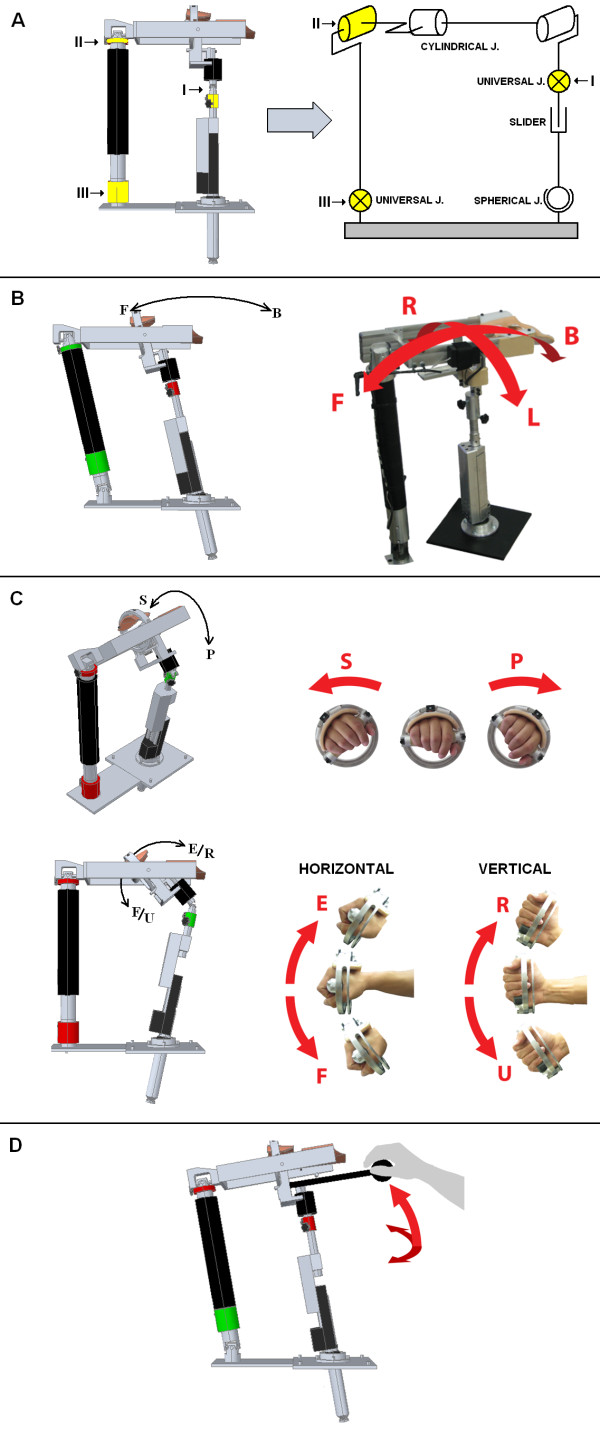
**A variable structure parallel mechanism**. A variable structure parallel mechanism enables using the device in different operational modes. Switching between modes can be easily achieved by locking or releasing joints I, II, III (A). Workspace in the "ARM" mode (B), "WRIST" mode (C) and "REACH" mode (D) are presented.

The main parts of the parallel mechanism are the three joints that can be either locked or released (Figure 2(A)). By locking or releasing DOFs in these joints (I, II, III), the mechanical configuration of VSP can be changed, enabling use of the device in several operational modes shown in Figure 2. The three possible modes ("ARM", "WRIST" and "REACH") are briefly described below.

"ARM" mode: locking joint I and releasing joints II and III, results in 2 DOF quasi-planar movements in Forward/Backward/Left/Right directions, as shown in Figure 2(B). The movement prescribed by the workspace "ARM" mode is similar to required for reaching and/or moving objects on a table, desk, or countertop.

"WRIST" mode: the mechanical configuration, termed as "WRIST" mode, is achieved by releasing joint I and locking joints II and III. A subject holding on the handle bar can perform movements in wrist as shown in Figure 2(C). By setting the offset orientation of the handle bar in the horizontal or vertical position, movement of all 3-DOFs in wrist (Flexion/Extension, Radial/Ulnar deviation and Pronation/Supination as shown in Figure 2(C)) can be achieved. The resulting movement of the user's wrist is similar to what is required for performing wrist-orienting motions in the following activities: pouring from a bottle, brushing teeth, or stirring a pot.

"REACH" mode: locking joints I and III and releasing joint II results in a mechanical configuration, which allows training of Forward and Up/Lateral reach movements. These motions are therefore similar to activities such as reaching for a high drawer or cupboard, or moving objects from one side of the cupboard to the other.

### Variable structure pantograph: series visco-elastic actuation

**Figure 3:**
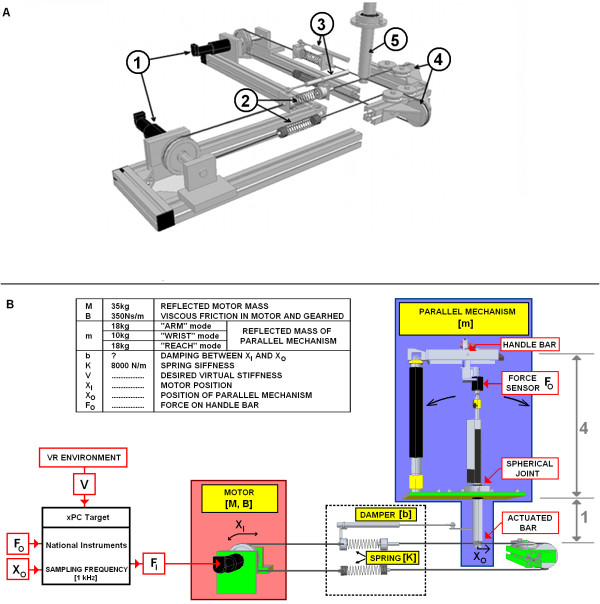
**Series visco-elastic actuator and parallel mechanism**. (A) Actuation of the VSP consists of: 1-two sets of DC motors with gears and encoders, 2-elastic springs, 3- mechanical dampers, 4-pulleys and 5-an actuated bar. Schematic presentation of the series visco-elastic actuator and parallel mechanism (only 1 DOF is shown for clarity), with characteristic values of mechanical component parameters used in the VSP. Impedance felt at the arm is 16 times smaller than at the bottom of the actuated bar, because the actuated bar is divided by a spherical joint in ratio 4:1.

The variable structure parallel linkage of VSP is actuated by a SEA based drive as shown in Figure 1. The implemented drive consists of two sets of DC motors (Maxon, RE40, 150 W) with gearheads (GP 52 C, 81:1) and incremental encoders. Torques from both gearheads' shafts impose force on the actuated bar through serially added mechanical springs and string wires, see Figure 3(A, B). The string wires are connected to the actuated bar perpendicular one to another, which enables actuation of the VSP in 2DOFs.

Introduction of an elastic element (mechanical spring) in series with the motor provides many benefits, including: more accurate and stable force control, attenuation of both backlash and friction nonlinear effects and the actuators' own impedance as well as providing greater shock tolerance (important for safety concerns). On the other hand, introducing SEA in haptic drive leads to reduction of the achievable force bandwidth. Since relatively slow movements can be expected during rehabilitation training, reduced force bandwidth does not present a significant problem.

Utilization of a variable structure parallel mechanism is essential in designing a versatile rehabilitation device. However, using a parallel mechanism where considerable endpoint mass is in series with both SEA's spring and motor mass, results in a coupled oscillator needing appropriate damping. Adequate dissipation of mechanical energy is needed, to achieve a stable haptic interaction when the device is in contact with a human. A convenient location for a mechanical damper may be in parallel with the SEA spring. Figure 3(A, B) presents a schematic illustration of an implemented parallel mechanism driven by a series visco-elastic actuator.

### Variable structure pantograph: linearized model

**Figure 4:**
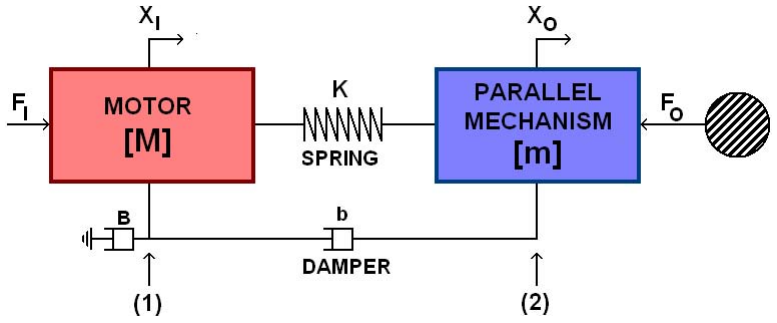
**A linearized model**. The motor with a gearhead (reflected mass M) is connected to the variable structure parallel mechanism (reflected mass m) via a spring (compliance K) and a viscous damper (damping B).

In Figure 4, the open loop model is illustrated, where M and m denotes masses, X_I _and X_o _positions, and F_I _and F_O _forces on the motor and the actuated bar, respectively. Attaching parallel mechanism on the actuated bar, significantly increases endpoint mass. The motor is coupled to the parallel mechanism via a mechanical spring K and damper b. The equivalent viscous friction in the motor and planetary gearhead is marked with B [17].

The relation between motor mass and the mass of the parallel mechanism can be given by two differential equations:

(1) $${\text{F}}_{\text{I}}\;-\text{M}{\ddot{\text{X}}}_{\text{I}}-\text{B}{\dot{\text{X}}}_{\text{I}}\;-\text{K}{\text{X}}_{\text{I}}\;+\text{K}{\text{X}}_{\text{O}}\;-\text{b}{\dot{\text{X}}}_{\text{I}}+\text{b}{\dot{\text{X}}}_{\text{O}}\;=0$$

(2) $$\text{K}{\text{X}}_{\text{I}}\;-\text{K}{\text{X}}_{\text{O}}\;+\text{b}{\dot{\text{X}}}_{\text{I}}\;-\text{b}{\dot{\text{X}}}_{\text{O}}\;-\text{m}{\ddot{\text{X}}}_{\text{O}}\;-{\text{F}}_{\text{O}}\;=0.$$

By taking Laplace transforms and relating equations, an expression relating F_O_, X_O _and F_I _can be found:

(3) $$\begin{array}{c} {\text{F}}_{\text{O}}=\left(-\frac{(\text{M}{\text{s}}^{2}+(\text{B}+\text{b})\text{s}+\text{K})(\text{m}{\text{s}}^{2}+\text{b}\text{s}+\text{K})-{\left(\text{b}\text{s}+\text{K}\right)}^{2}}{\left(\text{M}{\text{s}}^{2}+(\text{B}+\text{b})\text{s}+\text{K}\right)}\right){\text{X}}_{\text{O}}\\ +\left(\frac{\left(\text{b}\text{s}+\text{K}\right)}{\left(\text{M}{\text{s}}^{2}+(\text{B}+\text{b})\text{s}+\text{K}\right)}\right){\text{F}}_{\text{I}} \end{array}$$

This equation is important, for it determines the motor torque/force F_I_, needed to achieve a given output force F_O_, when the handle bar of the VSP is moving. If the parallel mechanism of the device is assumed to be clamped $\left({\dot{\text{X}}}_{\text{O}}=0\right)$, then the transfer function between output force and motor force is:

(4) $$\frac{{\text{F}}_{\text{O}}}{{\text{F}}_{\text{I}}}=\frac{\text{b}\text{s}+\text{K}}{\text{M}{\text{s}}^{2}+(\text{B}+\text{b})\text{s}+\text{K}}$$

On the other hand, the position to force transfer function of the uncontrolled plant (F_I _= 0) is defined as:

(5) $$\frac{{\text{F}}_{\text{O}}}{-{\text{X}}_{\text{O}}}=\frac{(\text{M}{\text{s}}^{2}+(\text{B}+\text{b})\text{s}+\text{K})(\text{m}{\text{s}}^{2}+\text{b}\text{s}+\text{K})-{\left(\text{b}\text{s}+\text{K}\right)}^{2}}{\left(\text{M}{\text{s}}^{2}+(\text{B}+\text{b})\text{s}+\text{K}\right)}$$

**Figure 5:**
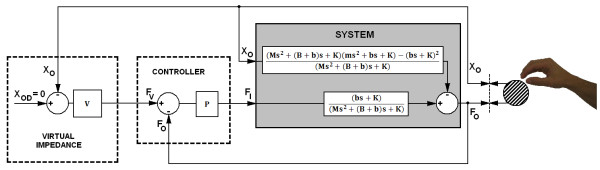
**Impedance based force control**. The inner force loop compares desired the virtual force F_V _with the force feedback F_O_. The output impedance loop calculates the desired virtual force F_V _from the position of the handle bar X_O_.V is the desired virtual stiffness and P the proportional gain of the controller.

The negative sign before X_O _comes from the definition of the directions of F_O _and X_O_. These two equations define the model of the plant to be controlled. The motion of the handle bar (X_O_) is modelled as a disturbance on the output force (F_O_), see shaded block in Figure 5.

The principal purpose of haptic devices is to allow human operators to touch, feel and manipulate objects in a virtual environment. For this reason, the impedance felt at the handle bar should be as close as possible to the desired virtual impedance (V, Figure 5).

Usually, three criteria are employed when designing haptic devices [19]: (1) movement in free space (LOW impedance) should be opposed with minimal possible force, (2) solid virtual objects (HIGH impedance) must feel stiff, and (3) virtual constraints must not be easily saturated, which requires a suitable impedance- based force control. In series elastic actuators, a variety of control strategies are possible. Williamson [20] proposed a control strategy for SEA with a feed-forward model and PID controller. Vallery [21] chose the concept of cascade force control with proportional-integral controller. We decided to implement the simplest approach, which is a proportional controller, in order to have a clearer picture on the influence of various mechanical components on passivity of haptic interaction [22,23].

Impedance based force control (Figure 5), was implemented in MATLAB (Simulink). In computer simulation, the VSP's haptic performance was investigated by simulating sinusoidal movements of the handle bar (X_O_). In simulation, LOW and HIGH impedance virtual force F_V _was compared to calculated force on the handle bar F_O_, for different values of damper b and proportional gain P. In order to investigate the option of using low-cost motors with potentially redundant backlash, different values of backlash were considered in the simulation model.

### Conditions regarding stability and passivity of the system

For a haptic system, the output impedance is usually defined as the transfer function from the velocity of the gripper, in our case handle bar $\left({\dot{\text{X}}}_{\text{O}}\right)$, to the opposing force (F_O_):

(6) $$\text{Z}(\text{s})=\frac{{\text{F}}_{\text{O}}}{-{\text{X}}_{\text{O}}\text{s}}.$$

Colgate [24] and Hogan [25] have proven that a system will be stable while in contact with changing environments if and only if the output impedance Z(s), obeys the following rules:

1. Z(s) has no poles in the right half plane

2. Re(Z(jw)) is nonnegative for all frequencies w

If these conditions are met, the impedance is a stable function of frequency and the system exhibits passivity.

By means of Equation 6 and simplified system model in Figure 5, output impedance of our controlled system is given by:

(7) $$\begin{array}{c} \text{Z}(\text{s})=\frac{{\text{F}}_{\text{O}}}{-{\text{X}}_{\text{O}}\text{s}}\\ =\left(\frac{(\text{M}{\text{s}}^{2}+(\text{B}+\text{b})\text{s}+\text{K})(\text{m}{\text{s}}^{2}+\text{b}\text{s}+\text{K})-{\left(\text{b}\text{s}+\text{K}\right)}^{2}+\text{V}(\text{b}\text{s}+\text{K})\text{P}}{\left((\text{M}{\text{s}}^{2}+(\text{B}+\text{b})\text{s}+\text{K})+(\text{b}\text{s}+\text{K})\text{P}\right)\text{s}}\right) \end{array}$$

First, we will check the condition of asymptotic stability. The characteristic equation for output impedance can be written as:

(8) $${\text{Ms}}^{3}+\left(\text{B}+\text{b}+\text{b P}\right){\text{s}}^{2}+\left(\text{K}+\text{K P}\right)\text{s}=0$$

It is obvious that Hurwitz determinants of the characteristic equation for Z(s) are nonnegative for all technically realizable values of mechanical components. Henceforth, Z(s) has no poles in right half plane and the first rule is met.

By replacing the complex variable "s" in Equation 7 by "jw", the frequency response Z(jw) can be obtained. The real part of the impedance frequency response is given by:

(9) $$\begin{array}{l} \text{Re}\left(\text{Z}(\text{j}\text{w})\right)=\text{Re}\left(\frac{{\text{F}}_{\text{O}}}{-{\text{X}}_{\text{O}}\text{j}\text{w}}\right)\\ =\left(\frac{\text{z}0+\text{z}2{\text{w}}^{2}+\text{z}4{\text{w}}^{4}}{{\left((\text{b}+\text{B}+\text{b}\text{P})\text{w}\right)}^{2}+{\left(\text{K}+\text{K}\text{P}-\text{M}{\text{w}}^{2}\right)}^{2}}\right) \end{array}$$

**Figure 6:**
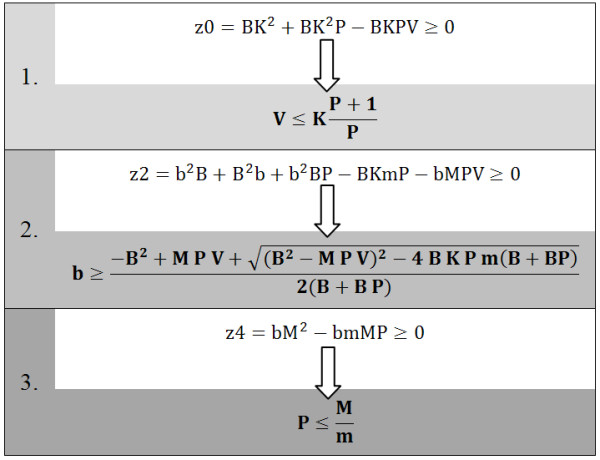
**Conservative conditions regarding passivity of the system**.

Re(Z(jw)) is nonnegative for all frequencies w, if all values z0, z2 and z4 are nonnegative (see Figure 6). This gives three conservative conditions for the passivity of the system that have to be considered.

First, the virtual stiffness V is limited by the stiffness of the mechanical spring K and controller's proportional gain P. However, if we look at the third condition, P is limited by the reflected motor mass M and reflected mass of the parallel mechanism m. It is straightforward, that value P can be increased by reducing m. The second condition demands that there must be sufficient damping between the relative position of motor X_I _and parallel mechanism X_O_. Usually, in technical realization of the system, damping is always present due to viscous friction in mechanical components but is not sufficient. For this reason, an additional damping element should be inserted in parallel with the spring to satisfy the damping condition for passivity of the system. The derived conservative conditions for passivity are general. In the following section, these conditions will be applied to characteristic values of the mechanical components used in technical realization of the VSP (listed in Figure 3(B)).

## Results

### Variable structure pantograph: application of derived passivity conditions

In the "ARM" and "REACH" mode of the VSP, estimated reflected mass of the parallel mechanism is relatively high (m = 18 kg). From the third condition on passivity, achievable controller's proportional gain is relatively small $\left(P\leq \frac{M}{m}≅1.9\right)$. This is due to the high reflected mass of the parallel mechanism m. From the first condition, maximal virtual stiffness V can be determined as $V\leq K\frac{P+1}{P}≅12000\;\;N/m$. Finally, from the second condition, damping of $\text{b}\geq \frac{-{\text{B}}^{2}+\text{M}\;\text{P}\;\text{V}+\sqrt{{\left({\text{B}}^{2}-\text{M}\;\text{P}\;\text{V}\right)}^{2}-4\;\text{B}\;\text{K}\;\text{P}\;\text{m}(\text{B}+\text{B}\text{P})}}{2(\text{B}+\text{B}\;\text{P})}≅\;\;800\;\text{N}\;\text{s}/\text{m}$ is needed, if we set virtual stiffness as **V = 12000 N/m **(solid virtual objects). On the other hand, if we want to emulate free space, where **V = 0 N/m **and all the other parameters remain the same, a much smaller damping of **b ≅ 250 Ns/m **satisfies the second condition for passivity.

In the "WRIST" mode, parameters needed to meet conditions of passivity are different. Given that almost all mass of the parallel mechanism is supported by joints II and III and therefore does not move (see Figure 2) and that only the segment with the handle bar is moving, the reflected mass is much smaller (m = 10 kg). For this reason values that satisfy conditions of passivity in the "WRIST" mode are different; see Table 1.

**Table 1:** Values of P, V and b that satisfy conditions regarding passivity.

	"ARM" and "REACH" mode [m = 18 kg]		"WRIST" mode [m = 10 kg]	
P	1,9		3,5	

V [N/m]	0 ≤ V ≤ 12000		0 ≤ V ≤ 10000	

	V = 0 N/m	V = 12000 N/m	V = 0 N/m	V = 10000 N/m
	
b [Ns/m]	250	800	210	800

It is obvious from Figure 6 that it is more demanding to meet conditions of passivity in the case where the end point mass (reflected mass of parallel mechanism m) is higher. For this reason, in further analysis higher mass of parallel mechanism (m = 18 kg in the "ARM" and "REACH" modes) was considered.

**Figure 7:**
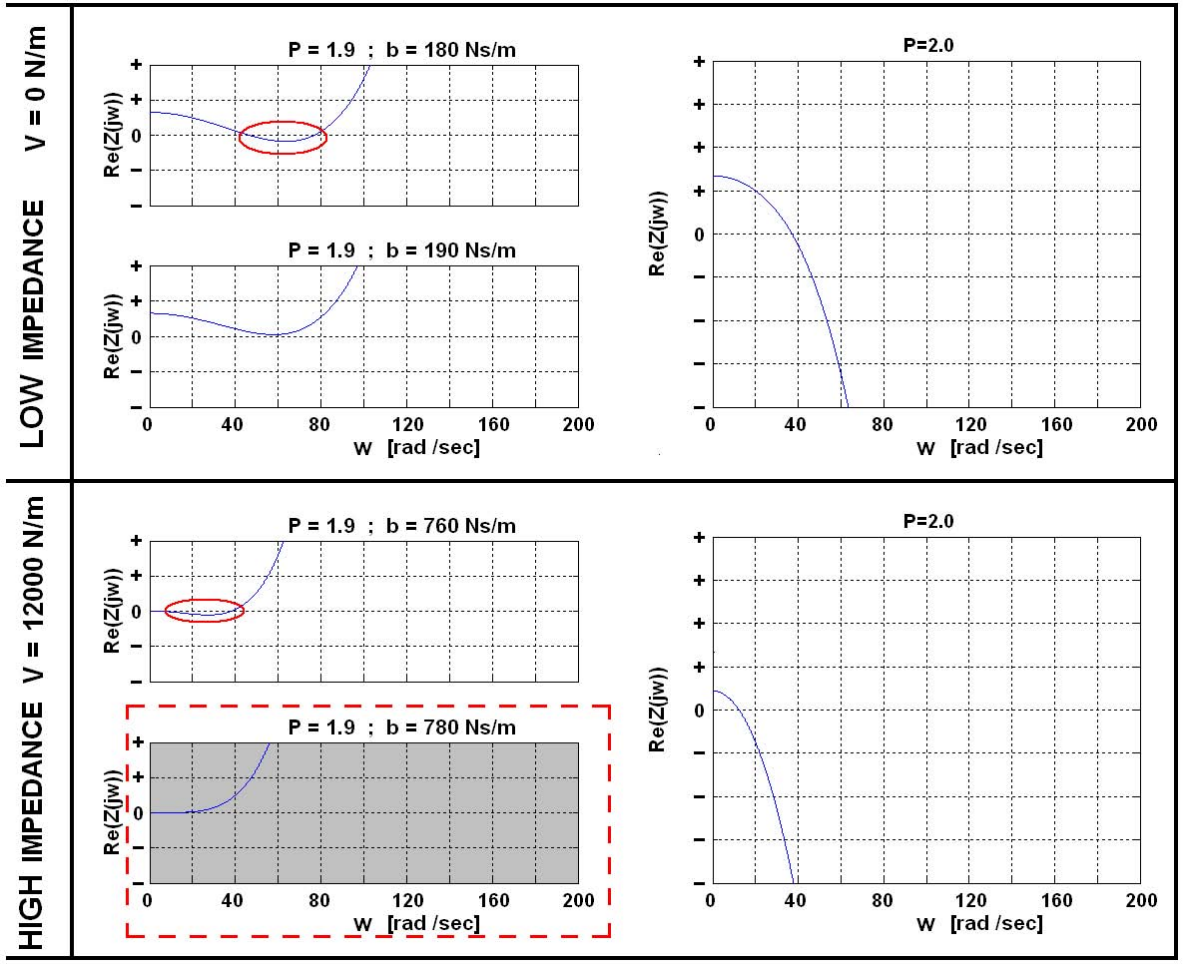
**System can exhibit passivity with suitable selection of P and b, for desired V**. If proportional gain of the controller P is higher than 1.9, Re(Z(jw)) becomes negative and therefore the system does not exhibit passivity. When emulating a LOW impedance environment (V = 0 N/m), damping of at least b ≥ 190 Ns/m is needed, while for a HIGH impedance environment, damping of at least b ≥ 780 Ns/m is needed.

Results listed in Table 1 present conservative conditions for passivity of the VSP, where all values z0, z2, and z4 are nonnegative. However, Re(Z(jw)) can be nonnegative for all frequencies w and desired V, also with suitable selection of P and b (see Figure 7). The maximal achievable virtual stiffness V in a HIGH impedance environment is 12000 N/m, which is sufficient for rehabilitation purposes. As can be seen from Figure 7, passive VSP behavior in any mode can be achieved (Re(Z(jw)) ≥ 0), if P ≤ 19 and parallel damping is at least b ≥ 780 Ns/m.

**Figure 8:**
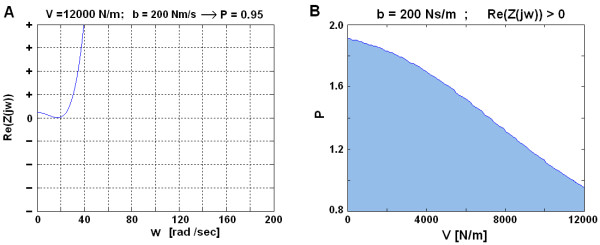
**By reducing virtual stiffness V, proportional gain P can be increased**. By reducing the controller' proportional gain to P = 0.95, parallel damping of b = 200 Ns/m is sufficient for the VSP' passivity when emulating a HIGH impedance environment (V = 12000 N/m) (a). However, P can be increased if a lower virtual stiffness V is desired (b).

When considering technical realization, a parallel damper with damping coefficient of b ≥ 780 Ns/m would be a rather heavy duty mechanical element. For this reason, it was decided to set b ≈ 200 Ns/m, which can be technically realized, however at the expense of reduced controller's proportional gain P. By demanding VSP' passivity in a HIGH impedance virtual environment (V = 12000 N/m) where parallel damping is b = 200 Nm/s, P should not exceed a value of 0.95 (see Figure 8(A)). By reducing the virtual stiffness V, proportional gain P can be increased (Figure 8(B), while maintaining VSP passivity.

### Variable structure pantograph: Simulation evaluation

Based on the results obtained in the previous subsection, simulation evaluation of VSP's haptic performance was undertaken (MATLAB, Simulink). In simulation model, a damper with b = 200 Ns/m was added parallel to the spring as depicted in Figure 3(B) and Figure 4. In terms of system passivity, the proportional gain of the controller P was varied from 1.9 in a LOW impedance to P = 0.95 in a HIGH impedance simulated environment. Haptic performance was investigated by simulating sinusoidal movements of the handle bar for ± 8 cm at frequencies of 1.0 Hz, 0.5 Hz and 0.1 Hz. It is important to point out that due to the design of the VSP (see Figure 3(B)), the displacement on the bottom of the actuated bar (X_O_) is 4 times smaller than the movement of the handle bar. Similarly, the force on the handle bar is 4 times smaller than the force on the bottom of the actuated bar where the cable wire is attached. For this reason, impedance felt by the subject holding the handle bar is 16 times smaller than on the bottom of the actuated bar. Therefore, the impedance felt by the user on the handle bar in a HIGH impedance simulated environment should be approximately 750 N/m (12000 N/m: 16) and the maximal force while repeating sinusoidal movements with amplitude ± 8 cm should be approximately 60 N (750 N/m * 8 cm). Desired force felt by the user in a LOW impedance simulated environment (0 N/m) should be 0 N. Additionally, the influence of backlash (1 mm and 4 mm), which is typically present in DC motors with planetary gears, was investigated.

**Figure 9:**
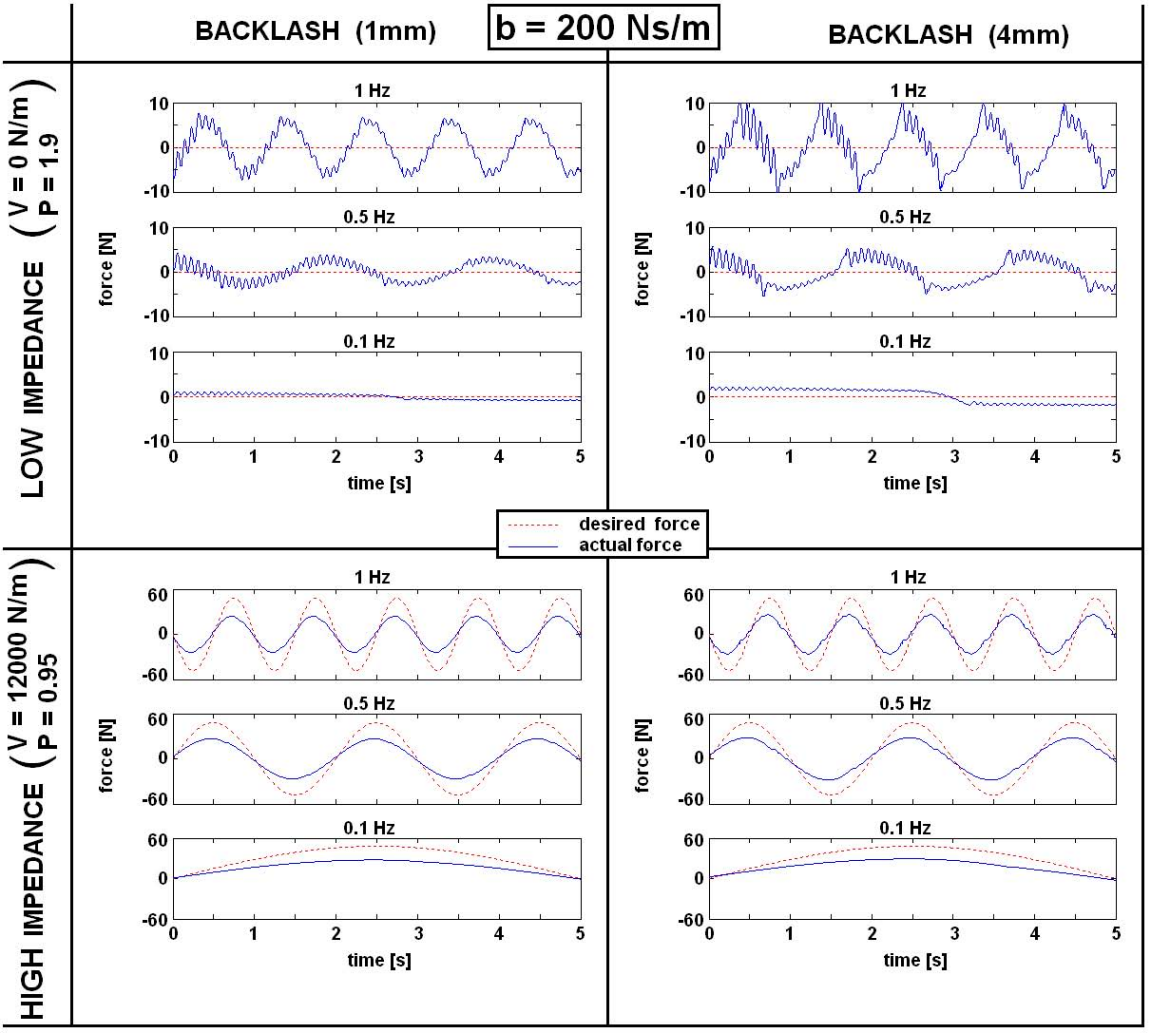
**Simulation evaluation**. Simulation of VSP performance in the ARM mode with a damper added parallel to the spring (b = 200 Ns/m) in LOW and HIGH impedance environments and for two values of simulated backlash (1 mm and 4 mm). Haptic performance was investigated by simulating sinusoidal movements (1.0 Hz, 0.5 Hz and 0.1 Hz respectively) of the handle bar X_O_, where the desired force (F_V_), was compared to the actual force (F_O_) on the handle bar.

Results of the VSP's haptic performance simulation with a parallel added damper (b = 200 Ns/m) are presented in Figure 9. The values of the forces presented in Figure 8 are interaction forces between the user and the handle bar and are approximately 4 times smaller than on the bottom of the actuated bar.

### Variable structure pantograph: experimental evaluation

**Figure 10:**
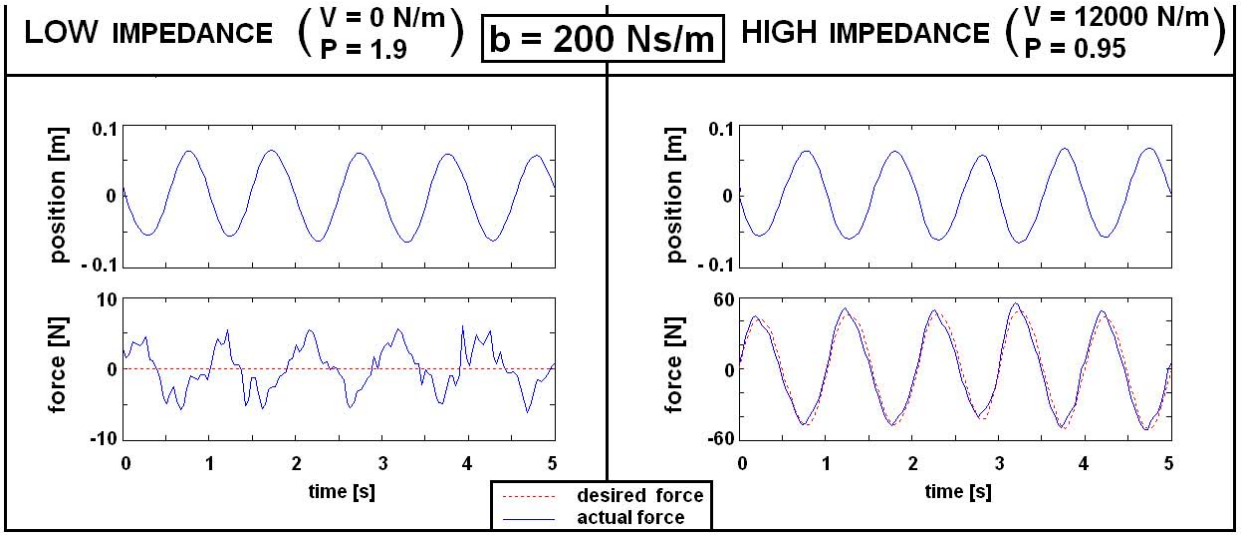
**Experimental evaluation of VSP performance in ARM mode with damper added parallel to spring (b = 200 Ns/m)**. Desired force is a product of the current position of the handle bar and desired virtual stiffness (V), divided by 16 due to the corresponding leverage implemented in the VSP design.

To verify results of the theoretical analysis and the simulation evaluation in previous subsections, the VSP's haptic performance was also experimentally examined on the recently developed VSP haptic robot [18]. Parallel to the spring, a damper with b = 200 Ns/m was added. The damper was technically realized by means of a pneumatic cylinder (FESTO), where damping was adjusted by setting appropriate air flow on the input and the output of the cylinder. Similar to simulation, the subject holding the handle bar imposed quasi-sinusoidal movements in the forward and backward direction for ± 8 cm at a frequency of approximately 1 Hz. Haptic performance was examined in the ARM mode by simulating LOW and HIGH impedance environments (see Figure 10). In a LOW impedance environment (V = 0 N/m), the actual interaction force between the user and the handle bar did not exceed 5 N, which is comparable to results from computer simulation. On the other hand, when simulating a HIGH impedance environment (V = 12000 N/m), the actual force corresponded to the desired values more precisely than predicted by simulation.

**Figure 11:**
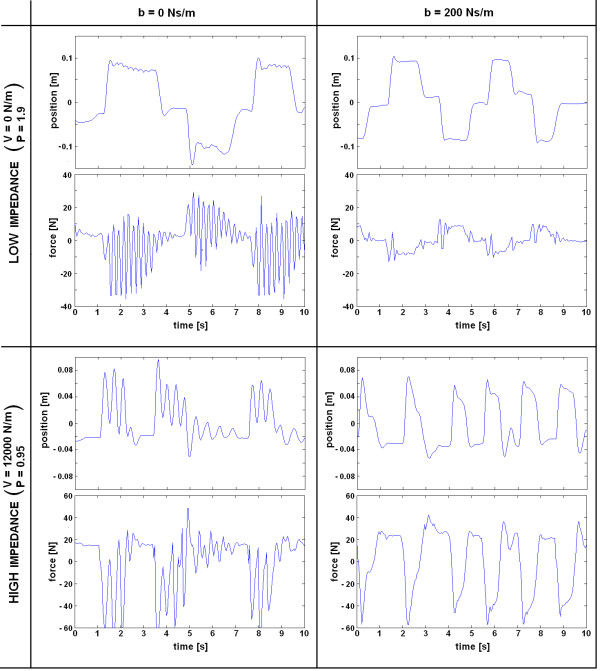
**VSP movement and interaction forces induced by fast movements of the handle bar**. Adding a damping element (b = 200 Ns/m) parallel to the mechanical spring significantly stabilizes haptic performance.

The passivity of the VSP was also tested experimentally. In order to destabilize haptic interaction between the human and the VSP, the handle bar was exposed to fast movements, without and with an added damper parallel to the spring. Haptic interaction in both cases is presented in Figure 11. It is obvious from Figure 11, that in the case when there is no damper added parallel to the spring, response of the VSP to fast movements (which normally do not occur in the rehabilitation movement) is much more oscillatory than in the case where a damper is added. In the oscillating interval, the subject is still holding the handle bar and thereby prevents the VSP from experiencing an unstable response. If the subjects were to release the handle bar during the oscillating interval, the VSP's response would become unstable, which could potentially lead to mechanical destruction of the device. This demonstrates that the VSP has stable behavior, if parallel to the spring, sufficient damping is presented. That was not the case when damper was omitted. Quantitative comparisons between simulations and experiments would not give meaningful results since the movement in the experimental evaluation is human-driven and therefore highly variable.

## Discussion

Actuators with series elasticity have been extensively studied in the field of robotics [14-16,20], where they were predominantly used in actuation of walking machines. Use of these actuators in haptic devices was limited to cases where the endpoint mass of devices are negligible as compared to the reflected inertia of the actuator [21], (included references reflect only a portion of the relevant literature). In case of the Variable Structure Pantograph haptic device, endpoint mass is not negligible due to a variable structure parallel mechanism. The main contribution of this paper is the derivation of passivity conditions that need to be fulfilled for a rehabilitation robot with a mechanism mass comparable to the reflected mass of the geared actuator. The results show that appropriate damping must be provided parallel to the SEA spring in order to obtain stable and passive behavior of the device when it is in contact with a human.

Three necessary conditions in terms of passivity were obtained from the theoretical analysis, which should be used when designing haptic devices with SEA actuation:

1.) The maximum proportional gain P of the controller must be limited by the ratio of the actuator and parallel mechanism reflected masses: $P\leq \frac{M}{m}$. Hence, better force control can be achieved (higher P) either by use of a motor with higher reflected inertia or by use of lighter parallel mechanism.

2.) The maximum achievable virtual stiffness V must be limited by controller's proportional gain P and the stiffness of mechanical spring K added in series to the motor: $V\leq K\frac{P+1}{P}$. This condition is similar to results obtained by Vallery [21], where it was shown that the SEA cannot display higher pure stiffness than the spring stiffness when passivity is desired.

3.) Third, to achieve haptic device passivity, sufficient damping b should be presented in parallel to the SEA spring: $\text{b}\geq \frac{-{\text{B}}^{2}+\text{M}\;\text{P}\;\text{V}+\sqrt{{\left({\text{B}}^{2}-\text{M}\;\text{P}\;\text{V}\right)}^{2}-4\;\text{B}\;\text{K}\;\text{P}\;\text{m}(\text{B}+\text{B}\text{P})}}{2(\text{B}+\text{B}\;\text{P})}$. Necessity of appropriate damping was also derived by Colgate and Schenkel [22], where the passivity of systems comprising a continuous time plant and discrete time controller was considered. This means that a damper inserted parallel to the spring ensures required dissipation of mechanical energy.

To verify results of theoretical analysis, simulation evaluation was undertaken. Simulation results predicted stable haptic performance for both HIGH and LOW impedance simulated environments. Simulation results revealed that haptic performance is also adequate in the case when higher values of backlash are presented in the system. This means that high-cost precision motors and gearheads that are currently used in VSP haptic device may be replaced by low-cost motors with greater backlash. Experimental evaluation has confirmed the simulation results and has shown that when appropriate damping and controller's proportional gain are used, stable interaction between machine and human are achieved in LOW and HIGH impedance environments, which was not the case when the damper was omitted. Generation of a HIGH impedance environment is limited to a virtual stiffness of 750 N/m, because the impedance felt at the arm is 16 times smaller than what the actuator can provide (12000 N/m:16). However, this does not present a notable limitation to rehabilitation where more compliant and thus gentle guiding in performance of training tasks is necessary. Also, experimental evaluation revealed that the achievable impedance range is sufficient [17].

In most cases described in literature, discrete linear models are used when dealing with general purpose sampled haptic environments, which are characterized with high Z-bandwidth [26,27]. It is important to point out that in this paper we utilized a continuous linear model of the studied haptic robot. In the particular case of the rehabilitation robot actuated with SEA, the typical Z-bandwidth is much lower (in our case the virtual stiffness is limited to 750 N/m). Also, the force bandwidth of the SEA as well as movement during rehabilitation are limited to app. 1 Hz [17], while the sampling rate is relatively high (1 kHz). Furthermore, it has been demonstrated that the effects of digitalization in conjunction with a usually high Z-bandwidth, (that a haptic interface should be able to render) can cause instabilities at frequencies of several hundred Hz, while at frequencies below 10 Hz, the effects of A/D and D/A devices placed within the control loop are negligible [27]. This enabled the use of a continuous linear model, which is much more intuitive to comprehend. The decision to model the parallel mechanism with a simple mass is related to the fact that the range of motion of the VSP is rather limited and relatively slow, meaning that the predominant dynamics will be dominated by the mass properties of the mechanism. Finally, the use of a linear model to mimic the dynamics of a geared DC motor has been experimentally validated in our previous paper describing the UHD robot [17].

## Conclusions

In conclusion the results of our study have shown that by properly designing rehabilitation device that uses a parallel mechanism and actuators with series elasticity, stability and passivity of haptic performance can be obtained. Because such a haptic system may be composed entirely of off-the-shelf mechanical components, versatile and affordable rehabilitation robotic devices can be produced, which may facilitate their wide spread use in clinical and home environments.

## Competing interests

University Rehabilitation Institute, Republic of Slovenia, received financial support for taking part in the development of the Variable Structure Pantograph device from FATRONIK Tecnalia, Spain. Both authors are co-authors of a patent application describing essential features of the Variable Structure Pantograph device while FATRONIK Tecnalia is the assignee of the same patent application.

## Authors' contributions

Both authors significantly contributed to the conception, theoretical analysis, simulation and experimental evaluation and writing of the manuscript. Both authors revised and approved the final manuscript.
